# Digital Push–Pull Driver Power Supply Topology for Nondestructive Testing

**DOI:** 10.3390/s25185839

**Published:** 2025-09-18

**Authors:** Haohuai Xiong, Cheng Guo, Qing Zhao, Xiaoping Huang

**Affiliations:** School of Resources and Environment, University of Electronic Science and Technology of China, Chengdu 611731, China; hawardbear@163.com (H.X.); guocheng@uestc.edu.cn (C.G.); zhaoq@uestc.edu.cn (Q.Z.)

**Keywords:** push–pull topology, digital signal control, pulse-width modulation (PWM), multi-stage MOSFET, fault protection, high-voltage power supply, nondestructive testing

## Abstract

Push–pull switch-mode power supplies are widely employed due to their high efficiency and power density. However, traditional designs typically depend on multiple auxiliary circuits to achieve functions such as power-up control, voltage regulation, and system protection, resulting in structural complexity and difficulty in debugging. Additionally, dual-power high-voltage amplifier systems often suffer from voltage deviations caused by supply imbalances or load fluctuations, potentially leading to equipment failure and significant economic losses. To overcome these limitations, we propose a novel digital signal-controlled push–pull driver power supply topology in this paper. Specifically, this design utilizes digital pulse-width modulation (PWM) signals to control multi-stage metal-oxide-semiconductor field-effect transistors (MOSFETs), incorporating adjustable duty-cycle drives, multi-channel current sensing, and fault protection mechanisms. Experimental validation was performed on a ±220 V, 20 kHz, 180 W power supply prototype. The results demonstrate excellent performance, notably enhancing stability and reliability in dual-side synchronous power supply scenarios. Thus, this digital-control topology effectively addresses the drawbacks of conventional push–pull designs and offers potential applications in nondestructive testing and high-voltage driving systems.

## 1. Introduction

Nondestructive Testing (NDT) techniques have become an essential tool widely utilized for structural health monitoring across various critical sectors such as aerospace, railway infrastructure, and energy engineering. Despite numerous existing studies exploring various NDT methodologies, the development of high-voltage power supplies specifically tailored for ultrasonic NDT systems remains relatively limited. Given their critical role in reliably driving piezoelectric transducers, advancements in high-voltage power supply technologies promise significant enhancements in detection sensitivity and operational efficiency [1]. Consequently, the research to optimize these power supplies is widely recognized as highly valuable and broadly applicable.

Currently, the existing literature addressing push–pull power supply topologies, although numerous, predominantly focuses on traditional analog-based control methods with significant reliance on auxiliary circuits. For instance, previous studies have emphasized improvements in efficiency and output stability through conventional push–pull methods [2]. However, these systems frequently encounter diverse challenges such as structural complexity, voltage instability, and inefficient asynchronous switching, ultimately restricting their practical deployment in precision-driven NDT contexts. In addition, the reliance on analog control strategies and auxiliary circuits continues to present notable drawbacks, including difficulties in debugging, limited adaptability under dynamic loads, and insufficient real-time monitoring capabilities [3,4]. Recent advances in digital power electronics have targeted these issues: Ghalebani et al. (2022) improved push–pull current regulation via digital peak control but lacked multi-channel sensing for NDT’s variable transducer loads [3]; Pidaparthy et al. (2015) achieved 92% efficiency at 20 kHz with digital push–pull control for LLC converters, yet failed NDT’s < 10 mV ultrasonic noise requirement [5]; Vivas et al. (2024) optimized high-voltage push–pull modeling but retained analog feedback, slowing response to NDT’s dynamic loads [6]. Additionally, Cvetanovic et al. (2023) proposed a median-based feedback filtering strategy to suppress switching noise and achieve very high control bandwidths in multi-sampled pulse-width modulated (MS-PWM) systems [7]. To address these limitations, it is necessary to extend prior research by incorporating digital control techniques to simplify the circuit complexity, enhance the operational stability, and provide much flexibility in handling the diverse load conditions.

In summary, a clear research gap remains in achieving a fully digital, multi-channel push–pull topology with integrated soft-start and protection for NDT. This work fills the gap by introducing a digital PWM-based design that simplifies circuitry, improves adaptability, and ensures reliable high-voltage operation. Therefore, this paper proposes a novel digitally controlled push–pull power supply topology to specifically design for the ultrasonic nondestructive testing applications. The proposed approach employs digital pulse-width modulation (PWM) to control multi-stage metal-oxide-semiconductor field-effect transistors (MOSFETs), integrating adjustable duty-cycle control, real-time multi-channel current feedback, and robust fault protection mechanisms. Experimental validation confirms that this digital approach significantly improves operational stability, reduces noise, and enhances multi-channel coordination, making it particularly suitable for advanced industrial scenarios requiring precise and reliable high-voltage power supplies.

## 2. Working Phase and Analysis of Current

**Figure 1:**
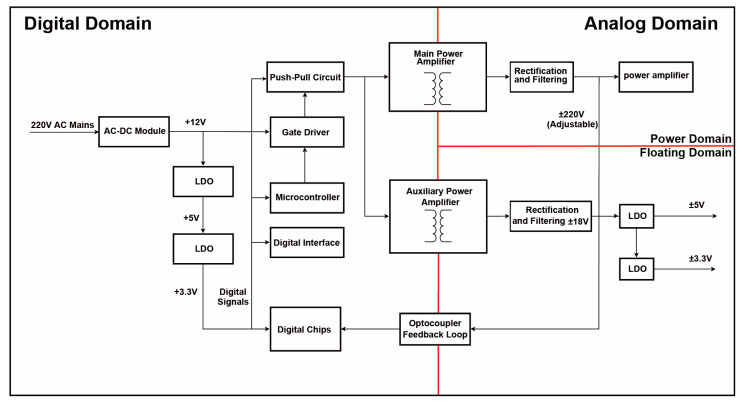
The power supply system used for the push–pull drive circuit.

The power supply system is critical in NDT and similar instruments, serving as the energy source for the entire system. As shown in Figure 1, this study details a power supply system designed for the push–pull drive circuit. The system uses a transformer for electrical isolation, dividing the design into two domains: the digital domain and the analog domain, with the analog domain further split into the power domain and floating domain. The digital domain handles control, signal processing, and data conversion, with a microcontroller generating PWM control signals to regulate system performance. A commercial AC-DC converter transforms the 220 V AC supply to 12 V DC, which powers the PWM signal and is stepped down to 3.3 V via low-dropout (LDO) regulators to supply the microcontroller and digital circuits [6,7,8]. The push–pull drive generates complementary PWM signals to control the transformer and regulate primary power output. The analog domain manages high-precision and real-time tasks like power conversion, filtering, and feedback control. It includes amplification, voltage/current sampling, and feedback circuits, ensuring fast response and low noise. The domain outputs ±220 V and ±18 V to supply the power amplifier and external devices, while isolated ±5 V and ±3.3 V supplies are used within the floating domain to reduce interference between digital and analog components.

**Figure 2:**
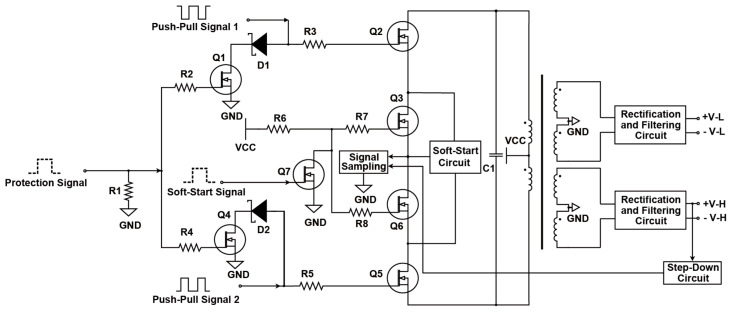
Digital push–pull driver power supply topology.

The proposed design improves the conventional push–pull power supply topology by integrating the soft-start, push–pull, sampling, and protection circuits via multiple digital control ports [9,10,11]. This allows precise control of the soft-start time, output voltage regulation, and real-time monitoring of current and voltage. Deviations trigger dynamic adjustments or shutdowns, ensuring system stability and safety. The topology, shown in Figure 2, generates a dual power outputs: a main power supply for high-power components and an auxiliary supply for analog system components [12,13]. The transformer’s input terminal is a critical node with high dV/dt. For instance, when the upper MOSFET Q2 transitions from ON to OFF while the lower MOSFET Q6 remains OFF, Q2’s drain voltage changes from 0 V to $2\cdot V_{CC}$, and Q6’s drain voltage drops from $2\cdot V_{CC}$ to 0 V [14]. Without a capacitor $C_{1}$ between these nodes, the voltage slope dV/dt is determined by dV/dt=I/C, where I is the transformer’s magnetizing current, and C is the parasitic capacitance, including MOSFET and PCB trace capacitances. In step-up transformers, the secondary dV/dt amplifies proportionally to the turns ratio (k), significantly increasing noise.

**Figure 3:**
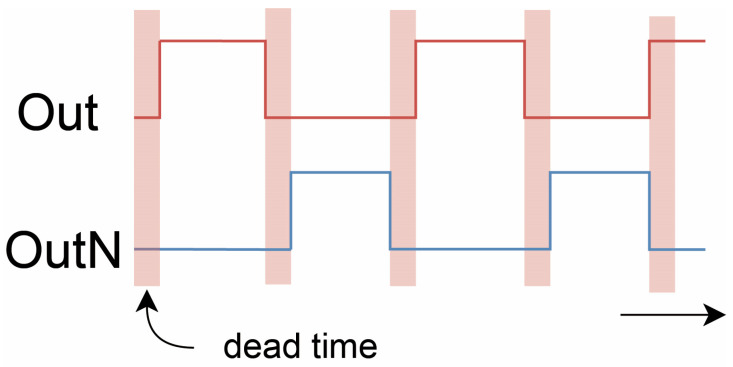
The dead time in switching power supplies.

To mitigate this, a capacitor is added between the MOSFET drains to increase node capacitance, replacing the parasitic capacitance and reducing dV/dt. The added capacitance slows the voltage transition, lowering noise. During this transition, both MOSFETs remain OFF simultaneously, introducing a necessary dead time, as illustrated in Figure 3. This approach effectively minimizes the high-frequency noise and stabilizes the circuit. By adjusting the duty cycle D, different output analog voltages can be obtained.

From the perspective of electromagnetic interference (EMI), reducing the dV/dt slope and carefully controlling dead time are also beneficial for electromagnetic compatibility (EMC). In conventional push–pull circuits, steep voltage transitions often generate high-frequency harmonics that couple into adjacent circuits and degrade the signal-to-noise ratio. The added drain–drain capacitance, combined with digital dead-time adjustment, effectively suppresses these harmonics and minimizes EMI emissions, which is particularly important for nondestructive testing applications requiring low-noise, high-stability operation.

The duty cycle is defined as the ratio of the high-level time $T_{1}$ to the total period $T_{1}$ The calculation formula is as follows [15,16]:

(1) $$D=\frac{T_{1}}{T}$$

The duty cycle D is influenced by controlling the duration of the dead time in the PWM signal. The relationship between the output voltage $V_{0}$ and the duty cycle is as follows [17]:

(2) $$V_{0}=\frac{V_{in}\times \frac{N_{S}}{N_{P}}}{2\times (1-D)}$$

The input voltage $V_{in}$, the primary winding turns $N_{P}$, and the secondary winding turns $N_{S}$ define the voltage transformation in the circuit. The gate drive signal originates from the PWM output of a microcontroller configured at 20 kHz with a 5 V high level, 0 V low level, and an adjustable duty cycle from 0% to 50%.

**Figure 4:**
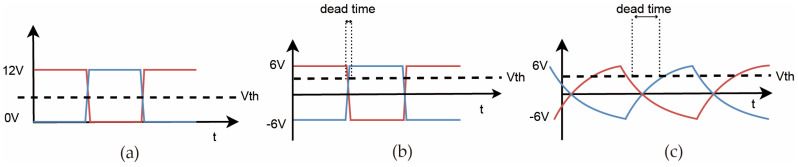
Gate drive voltage waveform before and after filtering schematic diagram: (**a**) Driver output waveform. (**b**) Waveform after high-pass filtering. (**c**) Final drive waveform after high-pass and low-pass filtering.

This signal is fed into an integrated gate driver, which amplifies the drive capability, inverts the signal, and converts it to 0 V–12 V, as shown in Figure 4a. The drive signals are then passed through CR high-pass filters to shift the range to +6 V to −6 V, as shown in Figure 4b. The inverted signals ensure that when one MOSFET conducts, the other is off, preventing short circuits in the transformer’s primary winding. Low-pass RC filters are then used to increase dead time when the duty cycle approaches 50%, as shown in Figure 4c, ensuring safe operation. To reduce noise and electromagnetic interference, the design minimizes the dV/dt at electrical nodes. High dV/dt slopes introduce high-frequency components that can propagate through the system, causing electromagnetic radiation and circuit interference. By optimizing the circuit design, these effects are mitigated, ensuring stable operation.

In the design of the push–pull driver circuit described in this paper, a soft-start circuit based on a PTC (Positive Temperature Coefficient) thermistor is introduced into the power supply system to prevent surge currents during startup, which could cause damage to circuit components or instability in performance. The soft-start circuit effectively limits the startup current by exploiting the temperature-dependent resistance characteristic of the PTC thermistor. As the temperature increases, the resistance rises sharply in the high-temperature range [18,19,20].

The resistance R(T) of the PTC thermistor typically follows the relation:

(3) $$R(T)=R_{0}\left(1+\alpha (T-T_{0})\right)$$

where $R_{0}$ is the base resistance, α is the temperature coefficient, and $T_{0}$ is the reference temperature. Near the Curie temperature, the resistance increases sharply and can be described using the following empirical formula:

(4) $$R(T)=R_{0}\exp\left(\frac{T-T_{c}}{\beta }\right)$$

where $T_{c}$ is the Curie temperature, and β is a material constant. During the initial startup phase, the PTC thermistor has a low resistance, and the current is relatively high. The current I(t) varies with time, following Ohm’s law:

(5) $$I(t)=\frac{V}{R_{\mathrm{circuit}}+R_{\mathrm{PTC}}(t)}$$

where $R_{\mathrm{PTC}}(t)$ increases with temperature. Once the circuit reaches a stable state, the current stabilizes, and the PTC thermistor reaches its maximum resistance $R_{\mathrm{PTC},\max}$:

(6) $$I_{\mathrm{stable}}=\frac{V}{R_{\mathrm{circuit}}+R_{\mathrm{PTC},\max}}$$

At this point, $R_{\mathrm{PTC},\max}$ can be expressed as [21,22,23]:

(7) $$R_{\mathrm{PTC},\max}=R_{0}\left(1+\alpha (T_{\mathrm{stable}}-T_{0})\right)$$

In the push–pull driver circuit design described in this paper, the PTC thermistor is connected in series between MOSFETs Q2 and Q5, and the soft-start circuit is activated by a digital signal to limit the surge current of the high-voltage power amplifier. When the power supply is turned on, the initial resistance of the thermistor is low, resulting in a high current. As the circuit operates, the resistance of the thermistor gradually increases, limiting the current rise [24,25,26]. This design effectively improves system stability and ensures smooth operation of the high-voltage power amplifier in nondestructive testing scenarios.

## 3. Circuit Design of Digital Push–Pull Drive Power Topology

Figure 2 illustrates the overall topology of the optimized digital-driven push–pull power supply, integrating a soft-start circuit, push–pull circuit, sampling circuit, and protection circuit. This design enhances the classic push–pull switching power supply by addressing voltage instability, noise interference, and dynamic load challenges.

As described in Equation (2), the precise output voltage regulation is achieved by adjusting the PWM duty cycle and the push–pull driver’s switching state. During load variations or changing output demands, the system dynamically adjusts the duty cycle based on real-time current and voltage feedback, maintaining stable output voltage.

Digital control improves duty cycle and dead-time regulation, addressing inefficiencies in traditional push–pull designs. Excessively short dead times can cause transistor overlap and short circuits, while long dead times reduce efficiency. Furthermore, high-frequency switching in conventional circuits increases dV/dt noise, destabilizing the power supply and interfering with surrounding circuits. To mitigate these issues, real-time sampling and protection mechanisms are implemented, improving dynamic response and overall stability.

The proposed topology features seven MOSFETs, with alternating conduction of Q2 and Q5 enabling efficient voltage conversion. A soft-start circuit (Q3, Q6, Q7) limits inrush current for smooth startup, while a protection circuit (Q1, Q4) rapidly responds to anomalies, enhancing reliability. Table 1 summarizes the key control circuits and their respective MOSFET states. This design demonstrates superior adaptability, stability, and performance under dynamic industrial conditions.

### 3.1. Soft-Start Circuit Design

The soft-start circuit is designed to mitigate large current surges during power supply startup. By controlling the duration of the gate voltage applied to MOSFET Q7, the circuit reduces inrush current, thereby protecting the components in the power supply.

**Figure 5:**
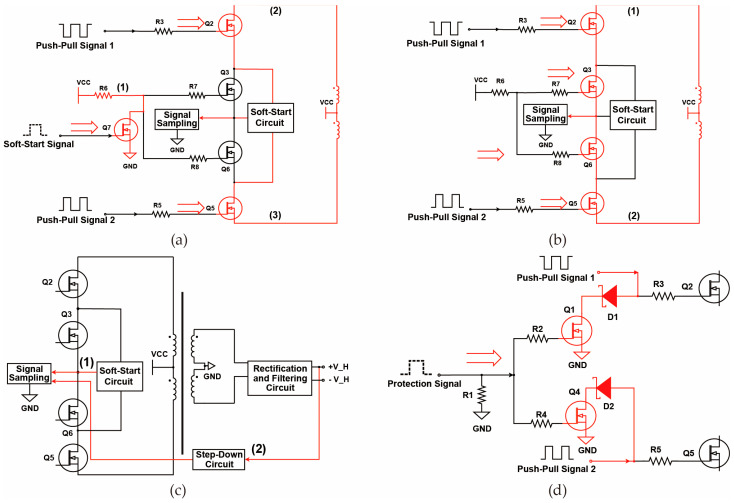
Circuit Design of the Digital Push-Pull Driver Power Supply Topology: (**a**) Soft-Start Circuit Design. (**b**) Push-Pull Circuit Design. (**c**) Sampling Circuit Design. (**d**) Protection Circuit.

Figure 5a shows the implementation of the soft-start circuit. The three curves in Figure 5a represent the current states at points (1), (2), and (3) in the power supply structure when the gate of MOSFET Q7 receives the soft-start signal. When the gate of Q7 is driven, Q7 turns on, and current flows from VCC to GND as indicated by the current direction (1). At this point, MOSFETs Q3 and Q6 remain off. Due to the push–pull signals, the gates of Q2 and Q5 are activated, causing Q2 and Q5 to conduct. The primary current then flows through the soft-start circuit in current directions (2) and (3), entering the signal sampling module and ultimately flowing to GND to complete the loop.

### 3.2. Push–Pull Circuit Design

As shown in Figure 5b, the push–pull circuit employs two PWM square wave signals Push–pull Signal 1 and Push–pull Signal 2—with a phase difference of 180°. By dynamically controlling the duty cycle of these signals, the circuit rapidly alternates the conduction and cutoff times of switching transistors Q2 and Q5, generating an alternating magnetic field that induces the required stable output voltage in the secondary winding.

When the Soft-Start Signal is deactivated, the push–pull circuit begins to generate two complementary square wave signals, with the direction of the current flow illustrated in Figure 5b. VCC applies a driving voltage to the gates of the field-effect transistors Q3 and Q6 through resistors R7 and R8, causing Q3 and Q6 to conduct. This action shorts the soft-start circuit, rendering it inactive. Subsequently, the signals driven by Push–pull Signal 1 and Push–pull Signal 2 are applied to the gates of Q2 and Q5, causing Q2 and Q5 to conduct. At this point, the current flows in the direction indicated by the arrows in Figure 5b into the signal sampling module and ultimately to GND, completing the circuit loop.

### 3.3. Sampling Circuit Design

As shown in Figure 5c, the sampling circuit is utilized to monitor the primary current and secondary output voltage in real time. By detecting the magnitude of the primary current, the system can determine whether the input current to the transformer is within normal operating ranges. Sampling the secondary output voltage serves a dual purpose: it not only assesses the operational status of the power supply system but also provides feedback to the preceding stage by comparing the actual output voltage with the target voltage. This feedback mechanism enables the control of the push–pull circuit to ensure that the output voltage meets the desired specifications and maintains dynamic stability.

In Figure 5c, arrows labeled (1) and (2) indicate the directions of the sampled currents during the normal operation of the power supply. When the power supply is activated, the primary current flows into the signal sampling module in the direction indicated by arrow (1). Simultaneously, the secondary voltage passes through the voltage reduction circuit and enters the signal sampling module in the direction shown by arrow (2). The sampling module then feeds this data back to the push–pull circuit and protection circuit to ensure the safety of the primary-side components and verify the normality of the current output.

### 3.4. Protection Circuit Design

As shown in Figure 5d, the protection circuit is designed to immediately shut down the power supply when an abnormal condition occurs, ensuring the safety of circuit components and users. In practical applications, when an abnormal output voltage is detected—such as a sudden increase in output voltage due to a short circuit in the backend circuit or a rapid rise in primary input current caused by damage to primary-side components—the signal sampling module feeds this abnormal information back to the preceding stage, triggering the protection signal. Specifically, as illustrated in Figure 5d, the arrows labeled (1) and (2) indicate the directions of current flow when the protection signal is activated. Upon activation of the protection signal, voltage is applied to the gates of Q1 and Q4, causing them to conduct. Push–pull Signal 1 and Push–pull Signal 2 pass through Schottky diodes D1 and D2, and then through Q1 and Q4 into GND. This action rapidly turns off the MOSFETs Q2 and Q5, cutting off power to the downstream circuit and thereby ensuring the safety of the power supply system.

## 4. Simulation Results

In order to evaluate the performance of the push–pull driver circuit, computer simulations of the current waveforms generated by the different circuit methods were performed in the LTSPICE (Version 24.1.10) circuit simulation software. The simulation parameters were set as follows: a switching frequency of 20 kHz, an input voltage of 12 V, a desired output voltage of 220 V, a desired output current of 1A, and a transformer primary-to-secondary winding ratio of 0.05:1. The parameters used in the simulation experiments are consistent with those in the actual experiments.

**Figure 6:**
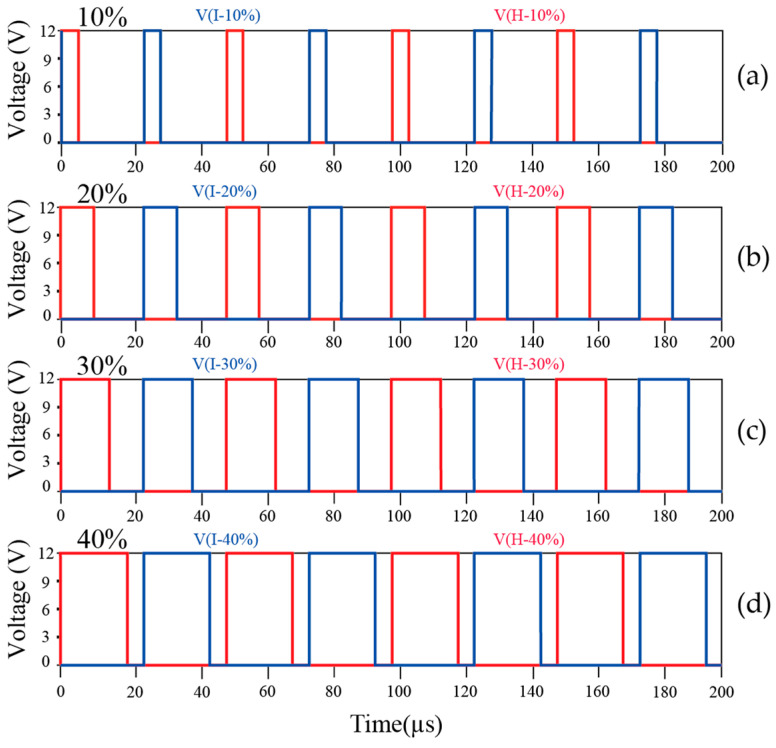
Simulated output waveforms of the push–pull circuit: (**a**) Duty cycle of 10%. (**b**) Duty cycle of 20%. (**c**) Duty cycle of 30%. (**d**) Duty cycle of 40%.

The first set of simulations tested the variation in the PWM duty cycle from 10% to 40% and the corresponding output voltage at a 40% duty cycle. By adjusting the duty cycle, the output voltage can be precisely controlled. The PWM waveforms at duty cycles of 10%, 20%, 30%, and 40% are shown in Figure 6.

**Figure 7:**
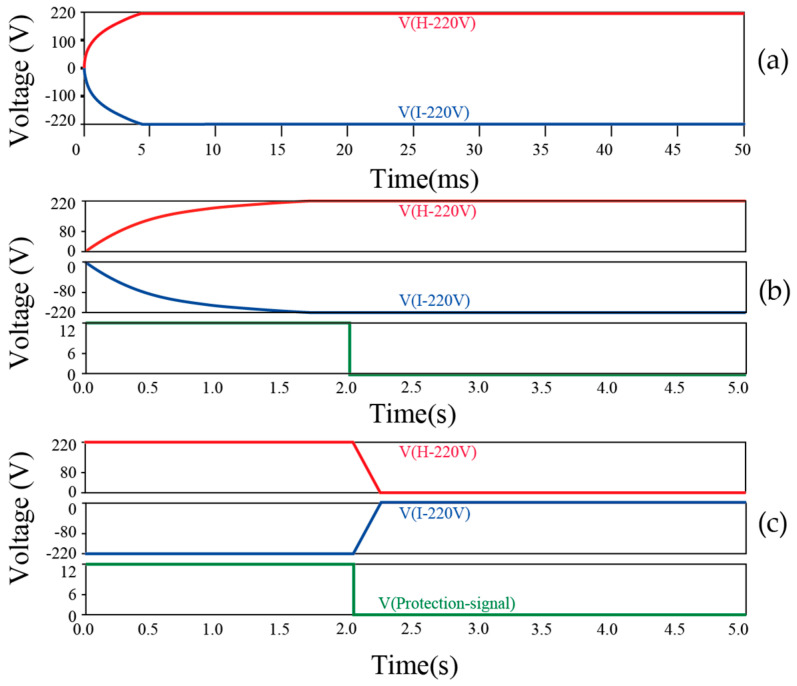
Simulation Results of output voltage behavior in the digital push–pull power supply system: (**a**) Output voltage waveform simulation at maximum duty cycle (±220 V) of the digital push–pull topology. (**b**) Soft-start process simulation: output voltage waveform and soft-start control signal. (**c**) Protection-triggered shutdown simulation: output voltage waveform and protection control signal.

At a duty cycle of 40%, the simulated waveforms of the dual-output voltage are shown in Figure 7a. The simulation results demonstrate stable output voltages of ±220 V, confirming the circuit’s effectiveness in meeting design specifications. The simulation of the soft-start circuit validated its ability to gradually increase the output voltage during startup and protect the system from inrush current impacts. As shown in Figure 7b, the simulation results indicate a slow voltage rise, confirming the effectiveness of the soft-start mechanism.

As shown in Figure 7c, the simulation results of the protection circuit signal control highlight its ability to respond promptly under abnormal conditions, such as output overvoltage or overcurrent. Upon activation of the protection signal, the power system rapidly shuts down, effectively safeguarding the system and its components, thereby demonstrating the robustness and reliability of the protection mechanism.

The simulations clearly demonstrate that duty cycle adjustment enables precise voltage regulation, as shown in Figure 6. The circuit maintains a stable ±220 V output at maximum duty cycle, as illustrated in Figure 7a. The soft-start mechanism achieves controlled startup without inrush current, as shown in Figure 7b. The protection circuit rapidly shuts down abnormal outputs to ensure system safety, as demonstrated in Figure 7c. These results confirm both stable voltage control and reliable fault protection.

By adjusting the PWM duty cycle, the circuit achieves precise voltage control. The soft-start mechanism ensures safe power-on operation, while the protection circuit provides rapid response under abnormal conditions. These features demonstrate that the proposed topology can effectively, flexibly, and reliably meet the demands for high-voltage, highly stable power supplies in nondestructive testing and other industrial applications.

## 5. Experimental Result

**Figure 8:**
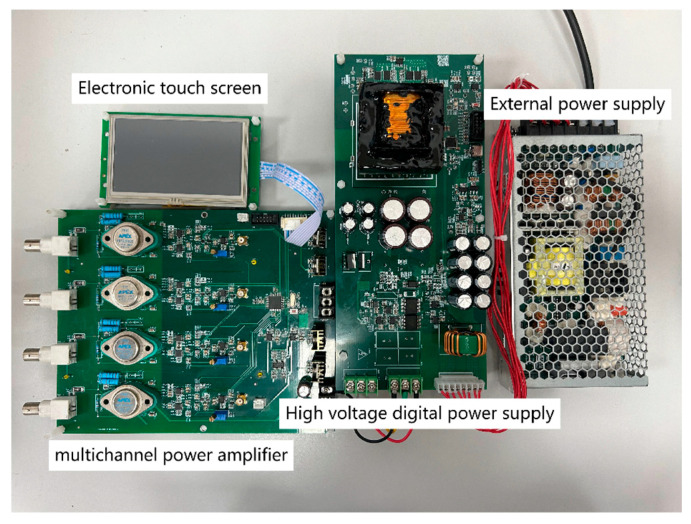
Four-channel high-voltage power amplifier designed based on the topology presented in this paper.

The experiment is carried out to validate the feasibility of the push–pull power circuit topology in practical applications by using a power supply system based on the proposed drive circuit design. The experimental parameters are configured as follows: a switching frequency of 20 kHz, an input voltage of 12 V, a target output voltage of 220 V, a target output current of 1 A, a transformer primary-to-secondary winding ratio of 0.05:1, and a duty cycle range of 0% to 45%. As shown in Figure 8, the power supply system is designed based on the proposed topology.

**Figure 9:**
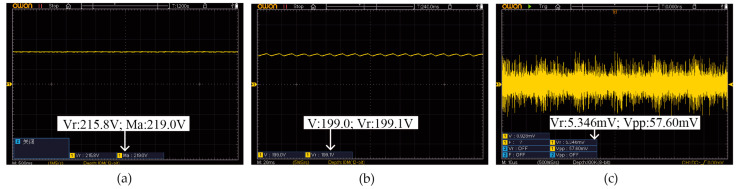
Voltage testing of the power supply system designed based on the topology presented in this paper: (**a**) Power output voltage under no-load condition. (**b**) Power output voltage under full-load conditions. (**c**) No-load noise of the power supply system.

The experiment initially evaluated the output waveform of the power system under both no-load and full-load conditions. As shown in Figure 9a, the output voltage waveform under no-load conditions demonstrates that the power supply maintains a stable output with no significant interference. Further measurements reveal that the no-load noise, as depicted in Figure 9c, is 5.346 mV, indicating good stability and low noise characteristics under no-load conditions. Under full-load conditions, the output voltage waveform is presented in Figure 9b. The experimental results indicate that the power supply successfully achieves the target voltage and current outputs while maintaining stable operation. Although the noise level during the experiment was influenced by environmental interference, the results validate the power system’s ability to maintain a stable output. These findings highlight the significance of the experimental validation, confirming that the proposed design not only meets performance specifications but also provides low-noise, stable operation suitable for high-precision applications. With future optimization of power supply design and control strategies, it is expected that the noise amplitude will be further reduced, thereby enhancing the system’s performance and reliability.

**Figure 10:**
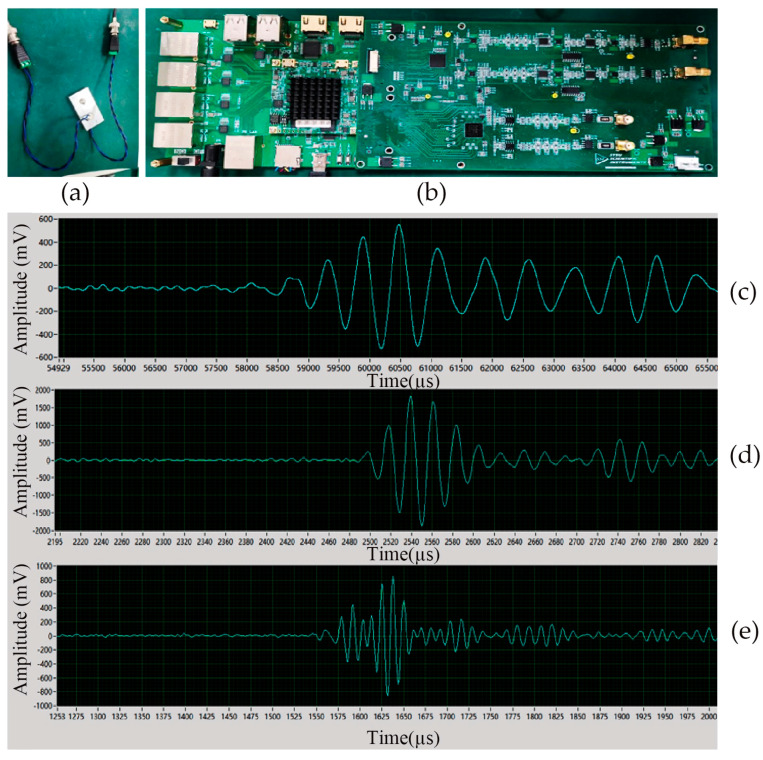
Experimental setup and echo images of ultrasonic testing using the upper computer: (**a**) Aluminum test sample. (**b**) Ultrasonic transmitter–receiver board. (**c**) Echo image collected for a 100 kHz five-peak wave input. (**d**) Echo image collected for a 300 kHz five-peak wave input. (**e**) Echo image collected for a 500 kHz five-peak wave input.

Next, using the laboratory’s ultrasonic piezoelectric transducer and aluminum test sample, as shown in Figure 10a, and the board, as shown in Figure 10b, the experiment tests whether the power amplifier powered by the push–pull drive circuit designed in this paper can successfully excite the piezoelectric transducer and collect the echo signal via the upper-level computer. A 4 Vpp five-peak waveform was output from the ultrasonic emission test board and amplified by the power amplifier powered by the push–pull drive circuit designed in this study. The result was a 100 Vpp Hanning-windowed signal used for excitation. The amplified excitation signal was applied to the test sample, with the frequency increased from 100 kHz to 500 kHz in separate experiments. Signals were collected at the receiving end using a data acquisition system.

The host computer, designed using LabVIEW software, provides functions including hardware connection control, parameter adjustment, and echo signal acquisition and display. The echo signal images at different frequencies, collected by the host computer, are shown in Figure 10.

The acquired echo images clearly demonstrate that the system successfully captures distinct five-peak waveforms across the frequency range of 100 kHz to 500 kHz. This indicates that the high-voltage amplifier, designed based on the Digital Push–pull Driver Power Topology, can effectively excite ultrasonic piezoelectric transducers and enable distortion-free echo signal acquisition throughout the entire operational bandwidth. The clarity and consistency of these signals emphasize the quality of the experimental validation, confirming that the proposed topology enables high-fidelity echo acquisition essential for reliable nondestructive testing. These results validate the feasibility and performance stability of the proposed topology. With further optimization of its design parameters and system integration, the Digital Push–pull Driver Power Supply Topology holds great potential for broader applications in radar sensing scenarios, such as short-range penetration detection and target identification, offering significant engineering value and practical applicability.

## 6. Conclusions

This paper presents a novel digital push–pull driver power supply topology to enhance the efficiency, stability, and adaptability of NDT systems. Traditional push–pull topologies, despite their efficiency, face challenges such as structural complexity, voltage instability under load variations, and limited protection mechanisms, restricting their application in high-precision testing. To address these issues, the proposed design incorporates digitally controlled multi-stage MOSFETs, PWM-driven adjustable duty cycles, real-time current detection, and fault protection for precise voltage regulation and improved reliability.

The topology includes a soft-start circuit to limit inrush currents, a push–pull driver for efficient power conversion, a sampling circuit for real-time monitoring, and a protection circuit for rapid anomaly response. Digital signal control enables flexible voltage adjustment, reduced dV/dt noise, minimized electromagnetic interference, and enhanced stability under load fluctuations. Simulations and experiments validate its performance, demonstrating a stable ±220 V output at 20 kHz switching frequency, precise duty cycle control, and effective mitigation of abnormal conditions. Practical tests with ultrasonic transducers confirm its capability to drive high-voltage amplifiers for NDT applications across a frequency range of 100 kHz to 500 kHz.

With superior efficiency, adaptability, and reliability compared to conventional designs, the proposed topology is ideal for industrial testing requiring multi-channel operation, stringent power supply for dual-rail amplifiers, and high-precision signal amplification. Future enhancements may extend its applications to radar sensing, power system testing, aerospace security, and advanced manufacturing.

## Figures and Tables

**Table 1:** Digital signal names and corresponding switch numbers for control circuits.

Circuit Name	Control Ports	Switches	Operation/Function
Push–pull Circuit	Push–pull Signal	Q2, Q3, Q5, Q6	Generates alternating drive through complementary PWM; Q3/Q6 bypass the soft-start path once deactivated.
Soft-Start Circuit	Soft-Start Signal	Q2, Q5, Q7	Activates Q7 to limit inrush current while Q3/Q6 remain off, enabling smooth startup.
Protection Circuit	Protection Signal	Q1, Q4	Turns on Q1/Q4 to ground PWM signals via diodes, rapidly shutting down Q2/Q5.

## Data Availability

Not applicable.

## References

[1] Mbele Ndzana N.D., Lekini Nkodo C.B., Tolok Nelem A., Pierre Pesdjock M.J., Abanda Y.A., Melingui A., Zeh O.F., Ele P. (2023). Contribution to the Development of a Smart Ultrasound Scanner: Design and Analysis of the High-Voltage Power Supply of the Transmitter. Inventions.

[2] Siji Das M., Jisha Kuruvilla D.B.P. (2013). Design, Theoretical Modeling, Simulation and Validation of a Push-Pull DC-DC Converter. Int. J. Adv. Res. Electr. Electron. Instrum. Eng..

[3] Ghalebani P., Teymoori V., Mwaniki F.M. (2022). Digital peak current mode control of isolated current-fed push-pull DC-DC converter with slope compensation. Int. J. Circuit Theory Appl..

[4] Zenk H. (2016). In push-pull converter output voltage stability comparison with using fuzzy logic, PI and PID controllers. Int. J. Eng. Res. Manag. IJERM.

[5] Pidaparthy S.K., Jang J., Choi B. (2015). Push–pull mode digital control for LLC series resonant dc-to-dc converters. IET Power Electron..

[6] Vivas F.J., Andujar J.M., Segura F. (2024). Non-ideal push–pull converter model: Trade-off between complexity and practical feasibility in terms of topology, power and operating frequency. Appl. Sci..

[7] Zeng Y., Lu G., Wang H., Du J., Ying Z., Liu C. (2014). Positive temperature coefficient thermistors based on carbon nanotube/polymer composites. Sci. Rep..

[8] Honarvar F., Varvani-Farahani A. (2020). A review of ultrasonic testing applications in additive manufacturing: Defect evaluation, material characterization, and process control. Ultrasonics.

[9] Xu Q., Wang H. (2022). Sound field modeling method and key imaging technology of an ultrasonic phased array: A review. Appl. Sci..

[10] Zang X., Xu Z.-D., Lu H., Zhu C., Zhang Z. (2023). Ultrasonic guided wave techniques and applications in pipeline defect detection: A review. Int. J. Press. Vessel. Pip..

[11] Lee C.-S., Shen Y.-T., Hsu W.-C., Huang Y.-P., You C.-Y. (2020). Al_0.75_ga_0.25_n/al_x_ga_1-x_n/al_0.75_ga_0.25_n/aln/sic metal–oxide–semiconductor heterostructure field-effect transistors with symmetrically-graded widegap channel. IEEE J. Electron Devices Soc..

[12] Zhou S., Wang J. (2021). An rf stress-based thermal shock test method for a cmos power amplifier. IEEE J. Electron Devices Soc..

[13] Petit P., Aillerie M., Sawicki J.-P., Charles J.-P. (2012). Push-pull converter for high efficiency photovoltaic conversion. Energy Procedia.

[14] Xiao J., Huo W., Yuan D., Liang C., Lai G., Li J., Xu H., Li S., Zhang S. (2024). A new pixel circuit for micro-light emitting diode displays with pulse hybrid modulation driving and compensation. IEEE J. Electron Devices Soc..

[15] Segovia Ramírez I., García Marquez F.P., Papaelias M. (2023). Review on additive manufacturing and non-destructive testing. J. Manuf. Syst..

[16] Singh G.P. (1986). Digital signal processing in ndt. NDT Int..

[17] Kim J.S., Javed K., Roh J. (2023). Design of a low-power and areaefficient ldo regulator using a negative-r-assisted technique. IEEE Trans. Circuits Syst. II Express Briefs.

[18] Kong Q., Chen H., Mo Y.-l., Song G. (2017). Real-time monitoring of water content in sandy soil using shear mode piezoceramic transducers and active sensing—A feasibility study. Sensors.

[19] George R., Ch N. (2024). A low-power 5-bit two-step flash analog-todigital converter with double-tail dynamic comparator in 90 nm digital cmos. J. Low Power Electron. Appl..

[20] Ning Z., Mao Y., Huang Y., Xi Z., Zhang C. (2020). A measurement noise rejection method in the feedback control system based on noise observer. IEEE Sens. J..

[21] Silapan P., Choykhuntod P., Kaewon R., Jaikla W. (2022). Duty-cycle electronically tunable triangular/square wave generator using lt1228 commercially available ics for capacitive sensor interfacing. Sensors.

[22] Pressman A. (1997). Switching Power Supply Design.

[23] Dokić B.L., Blanuša B. (2015). Power Electronics.

[24] Baliga B.J. (1996). Trends in power semiconductor devices. IEEE Trans. Electron Devices.

[25] Safonov E., Frolov V., Zhiligotov R., Petrenya Y. (2024). The specifics of ptc thermistor applications for limiting surge currents. Energies.

[26] Cvetanovic R., Petric I.Z., Mattavelli P., Buso S. (2023). Switching noise propagation and suppression in multisampled power electronics control systems. IEEE Trans. Power Electron..

